# Advancing Age May Decrease Mitochondrial Activity in Cumulus Cells

**DOI:** 10.3390/jcm13102800

**Published:** 2024-05-09

**Authors:** Suwichaya Jitngamsujarit, Lingling Salang, Charupong Saengboonmee, Supannika Sorin, Kanyarat Thithuan, Thanida Pongsritasana, Sineenart Sukkasame

**Affiliations:** 1 Department of Obstetrics and Gynecology, Faculty of Medicine, Khon Kaen University, Khon Kaen 40002, Thailand; suwichaya.j@gmail.com (S.J.); pthani@kku.ac.th (T.P.); tangmohp@gmail.com (S.S.); 2 Department of Biochemistry, Faculty of Medicine, Khon Kaen University, Khon Kaen 40002, Thailand; charusa@kku.ac.th (C.S.); s_supannika@kkumail.com (S.S.); kanyarat.th.60@ubu.ac.th (K.T.)

**Keywords:** advanced age, cumulus cells, mitochondrial activity, mean fluorescence index, blastocyst quality

## Abstract

**Background:** The goal of this study was to compare mitochondrial activity in cumulus cells (CCs) between young and advancing-aged women, the factors that affect mitochondrial activity, and their association with blastocyst quality. **Materials and methods:** This prospective study included 80 infertile women who underwent ICSI between May and October 2023. Participants were divided into two groups: older and younger than 38. The oocyte mitochondrial activity from CCs was evaluated using MitoTracker, and the mean fluorescence intensity (MFI) was also evaluated. **Results:** The univariate and multivariate analyses revealed a significant difference in the MFI between the woman ≥ 38 age group and the lower age group (162.68 ± 79.87 vs. 228.39 ± 121.38; *p*-value = 0.005; 95%CI 19.97, 111.45). The factors that affected the MFI were women ≥ 38 years of age (*p*-value = 0.005; 95%CI −111.45, −19.91), total gonadotropin dosages (*p*-value = 0.006; 95%CI −0.08, 0.01), and gonadotropin-releasing hormone agonist (GnRHa) triggering (*p*-value = 0.006; 95%CI 36.46, 210.06). However, only women aged ≥38 years remained statistically significant after a multivariable regression analysis (*p*-value = 0.014; 95%CI −121.00, −14.30). In addition, only male age (mean age ± SD = 38.26 ± 5.13) was associated with high blastocyst quality in univariate and mixed multivariate analyses (OR 0.91; 95%CI 0.56, 3.04). The chemical pregnancy rate was not significantly different between the two age groups (34.5% vs. 56.7%; *p*-value = 0.162; 95%CI 0.2, 1.30). **Conclusion:** Advancing age decreased mitochondrial activity in CCs but did not affect blastocyst quality. By contrast, male age may be a predictor of high-grade blastocyst quality.

## 1. Introduction

The aging process involves almost all vital organs, including the brain, heart, and lungs [1]. The female reproductive system is susceptible to age-related changes [2]. Female fertility peaks at age 25 and declines after 37 years of age [3]. Reproductive aging causes changes such as follicle atresia and apoptosis [4], but the impact on somatic cells and oocytes remains unknown. Depletion of mitochondria and increased point mutations are the hallmarks of aging [5]. A decrease in mitochondria impacts intracellular energy production and tissue development [6]. 

Oocytes contain the highest concentration of mitochondria and copies of mitochondrial DNA [7]. The number of mitochondrial copies increases with oocyte development. Primordial germ cells contain few mitochondria, and their number increases significantly during the antral stage [8]. Cumulus granulosa cells (CCs), which are granulosa cells (GCs) that encircle the oocyte, increase the number of mitochondria in the antral follicle stage in order to generate energy for self-development and oocyte development [9,10,11]. Granulosa and theca cells regulate mechanisms involved in oocyte maturation [12]. To preserve normal follicular development, these cells undergo a highly differentiated process that involves various paracrine interactions [13]. CCs have high glycolytic activity, unlike oocytes, which have poor glucose metabolism. ATP and energy from CCs supply oocytes through gap junctions [10]. Hence, mitochondrial dysfunction in CCs may impair oocyte maturation and embryo development. CCs could be the best noninvasive test for oocyte quality [14].

Studies have found that quantity, DNA copy number, and DNA content in the mitochondria of CCs affect oocyte maturation, fertilization, early embryonic development in mice and humans, and embryo implantation [15,16,17,18,19]. In contrast, some studies have found no association between DNA content and oocyte maturation or blastocyst formation [20]. 

Advancing age is inversely related to mitochondrial function in CCs. Women at age 38 had rapidly declining follicles and ovarian reserves [21,22]. The study found that women also had decreased mitochondrial DNA copy numbers, and women in this group had increased mitochondrial DNA deletions, affecting embryo quality [23]. Moreover, women over 38 had lower mitochondrial activity, number, and ATP quantity [14,24,25].

Most current studies have focused on the copy number of mitochondrial DNA in CCs. Few studies have investigated mitochondrial activity on the clinical outcomes. This study aimed to find out the mitochondrial activity in aging women, the factors affecting mitochondrial activity, and the factors associated with blastocyst quality.

## 2. Materials and Methods

### 2.1. Study Design

The sample size was determined based on the findings of our pilot study, which indicated that an average difference between the two groups was 254.58 ± 180.5 and 152.1 ± 95.2, respectively. We specified a type I error of 0.05 and 80% power. The current study recruited 80 participants with provision for a 20% dropout rate.

This prospective cohort study was conducted between May 2023 and October 2023 at the Infertility Clinic of Srinagarind Hospital, Faculty of Medicine, Khon Kaen University, Thailand. The Khon Kaen University Ethics Committee for Human Research, Thailand (HE661193), reviewed and approved the study. All participants were counseled, and written informed consent was obtained from them.

### 2.2. Study Population

Infertile women undergoing controlled ovarian hyperstimulation (COH) at the Infertility Clinic, Srinagarind Hospital, were screened, recruited, and divided into two groups: under 38 and over 38. Women under 38 were defined as the young age group, while those over 38 were defined as the advancing-age group. Information was obtained regarding demographic characteristics, obstetrical, medical, surgical history, and prior fertility treatment. Physical examination, ultrasound of pelvic organs, and blood testing were performed at first visit. Normal ovarian reserve was defined by antral follicle count (AFC) ≥ 5. All male partners were examined and investigated (semen analysis).

### 2.3. Intervention

All participants underwent intracytoplasmic sperm injection (ICSI) with an antagonist or progestin-primed ovarian stimulation (PPOS) protocol. The doctor individually assigned the gonadotropin dose, formulation, and trigger drugs. After the oocytes were picked up, CCs were collected, cultured, and dyed with MitoTracker Red CMXRos. The accumulation of mitochondria stained with red fluorescent dye in living cells was determined by membrane potential. The mean fluorescence intensity (MFI) was measured and reported. This can express the mitochondrial activity.

### 2.4. Ovarian Stimulation Protocol

The participants under 38 years of age received rFSH (Follitrope^®^: LG Chem, Jeollabuk-do, Korea, or Gonal F^®^: Merck-Serono, Geneva, Switzerland, or Puregon^®^; Organon Oss, Netherlands), while the other group used rFSH + hp-hMG (Menopur^®^: Ferring Pharmaceutical, Copenhagen, Denmark, or Follitrope^®^ plus IVF-M^®^; LG, Jeollabuk-do, Korea), or rFSH + rLH (Gonal F^®^ plus Pergoveris^®^: Merck-Serono, Geneva, Switzerland) for ovarian stimulation. Cetrorelix (Cetrotide^®^; Merck-Serono, Geneva, Switzerland), Ganirelix (Orgalutran: Organon^®^, Oss, Netherlands), progesterone (Utrogestan^®^; Besins Healthcare by OLIC, Ayutthaya, Thailand), or medroxyprogesterone acetate (Provera^®^ Pfizer Italia S.r.l., Ascoli, Italy) were used for pituitary gonadotropin suppression. In each case, ovulation was induced with a single trigger (recombinant HCG; Ovidrel^®^; Organon Oss, Netherlands, or gonadotropin-releasing hormone agonist (GnRHa); Decapeptyl^®^: Ferring Pharmaceutical, Copenhagen, Denmark) or dual trigger (Ovidrel^®^; Organon Oss, Netherlands, plus Decapeptyl^®^: Ferring Pharmaceutical, Copenhagen, Denmark). The oocytes were retrieved transvaginally using ultrasound guidance. The data are presented in Table 1.

### 2.5. CCs Collection Methods

Oocyte–cummulus complexes (COCs) were washed with FertiCultTM Flushing medium (Fertipro, Beernem, Belgium) and cultured in fertilization medium (G-TLtm, Vitrolife, Gbg, Sweden) in a CO_2_ incubator (37 °C, 5%CO_2_, and 95% humidity) for 4 h. The oocytes were stripped using chemical (hyaluronidase 80 IU/mL; SAGE MediaTM, Origio, Malov, Denmark) and mechanical techniques using pipette with a diameter of 190 µm (Sunlight Medical, Inc., Jacksonville, FL, USA). The denuded oocytes were suctioned using a pipette with a diameter of 130 µm (Sunlight Medical, Inc., Jacksonville, FL, USA) and incubated for the ICSI process. All CCs separated from each oocyte were collected in 15 mL EP tubes to evaluate mitochondrial number.

### 2.6. Quantified Mitochondrial Activity Using MitoTracker Staining

**Figure 1:**
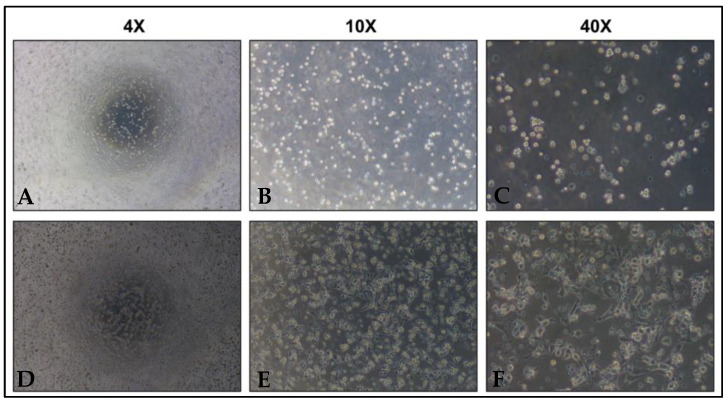
Case examples of CCs under microscope. (**A**–**C**) CCs before incubation. (**D**–**F**) CCs after incubation for 16–18 h.

Cells were washed with 1X phosphate buffer saline (PBS) at pH 7.4, then centrifuged at 1500 rpm at 4 °C for 5 min twice. The cell pellet was then re-suspended in 500 µL DMEM-HG (Thermo Fisher Scientific, Waltham, MA, USA) and supplemented with 10% FBS. Finally, the cell suspension was plated onto 48 well plates. The cell fixation plates were incubated at 37 °C in 5% CO_2_ for 16–18 h (Figure 1).

**Figure 2:**
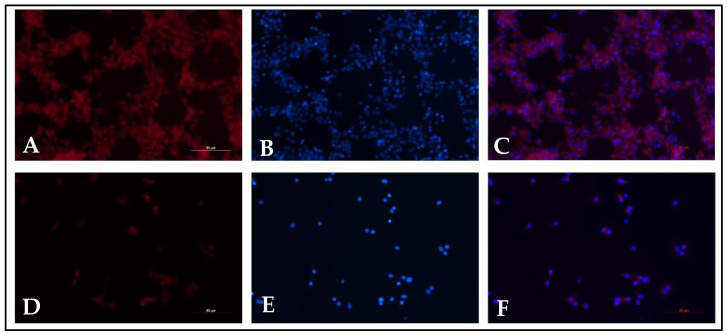
CCs under MitoTracker. (**A**–**C**) CCs with high MFI. (**D**–**F**) CCs with low MFI. (**A**,**D**) CCs with MitroTracker red dye staining. (**B**,**E**) CCs with Hoechst. (**C**,**F**) CCs with merge staining.

The MitoTracker was prepared at a concentration of 1:500 (Thermo Fisher Scientific, Waltham, MA, USA) in DMEM-HG media and replaced with media containing MitoTracker to adhere cells, which were incubated at 37 °C and 5% CO_2_ for 15 min. The medium was disinfected, and the cells were washed with PBS 2 times. Cells were fixed with 4% paraformaldehyde (Merck Schuchardt OHG, Hohenbrunn, Germany) on ice for 15 min, discarded, and washed with PBS 2 times. Nucleus cells were stained with 1:10,000 Hoechst (Eugine, OR, USA) in PBS for 30 min in a dark box, discarded, and washed with PBS 2 times. Cells were fixed with 4% paraformaldehyde (Merck Schuchardt OHG, Hohenbrunn, Germany) on ice for 15 min, then discarded and washed with PBS 2 times. Nucleus cells were stained with 1:10,000 Hoechst (Eugine, OR, USA) in PBS for 30 min in the dark box, then discarded and washed with PBS 2 times. The stained cells were kept free from light at 4 °C. The cells were examined using a fluorescence imaging microscope/Tiu, and five areas were captured (Figure 2). Mitochondrial activity was quantified from the MFI using the ImageJ 1.53k software. The MFI was calculated from the fluorescence signal of MitoTracker divided by the number of cells in each field.

### 2.7. ICSI/Embryo Culture/Embryo Grading

ICSI treatment was initiated for all women, and all embryos were cultured using Vitrolife Embryoscope^®^. Vitrification technology was used for embryo cryopreservation under the standard procedures of Srinagarind Hospital. The Istanbul consensus document was followed in assigning a score to embryos based on the degree of blastocyst expansion, inner cell mass (ICM), and trophectoderm (TE) grade demonstrated [26].

### 2.8. Statistical Analysis

Means and standard deviations were used to characterize the continuous variables. The independent *t*-test was used for normally distributed data to compare continuous variables, and the Mann–Whitney test was performed when the distribution was not normal. The Chi-squared (χ^2^) and Fisher’s exact test were used as appropriate for all binary and qualitative variables to test the differences in proportion. All analyses were performed using STATA version 16.

The association between outcomes (age, MFI, fertilization, total dose of gonadotropin, type of ovarian stimulation, good embryo quality, and male factor) and the studied factors were analyzed using univariate and multivariate logistic regression. Mixed-effect linear regression was used to account for the cluster of oocytes of individual participants (fertilization and good embryo quality). Statistical significance was defined as *p* ≤ 0.05. A 95% confidence interval (CI) of the mean difference (MD) was used to analyze the precision of the data.

## 3. Results

### 3.1. Participant Flow

**Figure 3:**
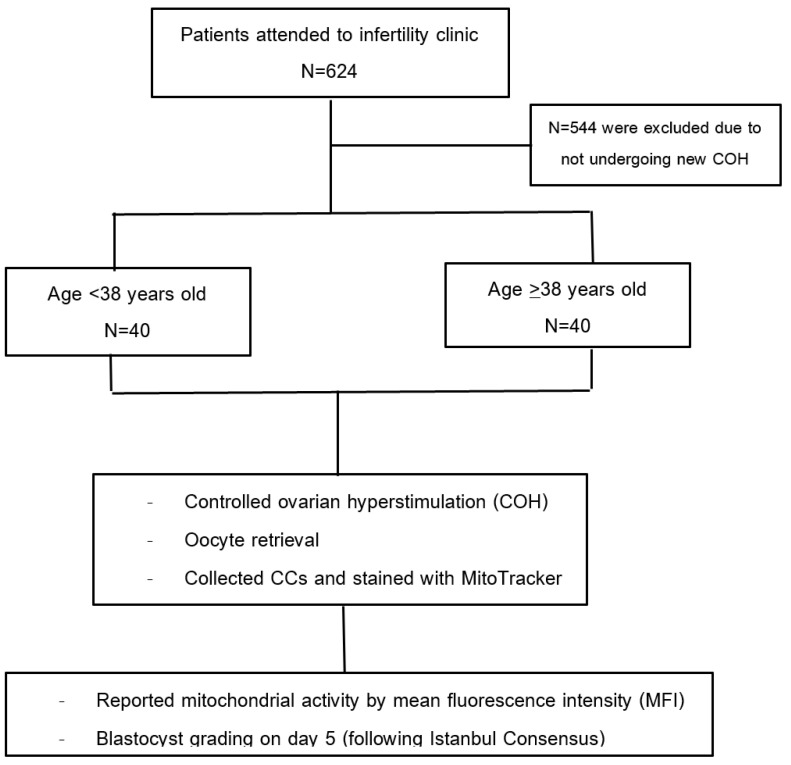
Study flow.

In total, 624 patients were screened, and 554 were excluded because they did not undergo a new COH (Figure 3). Eighty participants were enrolled in the study. All participants completed the study.

### 3.2. Baseline Characteristics

The participant characteristics and demographic data are summarized in Table 1. As expected, the mean age was substantially lower because these variables were used to categorize the patients into young age (YA) and advancing-age (AA) groups. The mean age of the young group was 32.9 ± 2.5 years, and the other group showed a mean age of 40.9 ± 2.3. The average male age was also significant between the two groups (37.4 ± 5.4 vs. 42.3 ± 5.0; *p*-value < 0.001). BMI, infertility factors, duration of infertility, total dosage of gonadotropin use, and ovarian triggers were compared between the two groups. There were no significant differences in BMI, type, cause, or duration of infertility. The antral follicle counts (AFC) in the AA group were significantly lower than the YA group (4.55 ± 2.20 vs. 9.15 ± 4.35, *p*-value < 0.001). Presumably, the total FSH dosages administered during the stimulation phase were greater in the AA than the YA group, with statistical significance. Nonetheless, there was no discernible variation in the use of medications for ovarian stimulation or LH suppression between the two groups.

The mean MFI was significantly higher in the YA group than in the AA group (228.39 ± 121.38 vs. 162.68 ± 79.87, *p*-value = 0.005; 95%CI 19.97 to 111.45). In addition, the YA group had a substantially higher mean of the number of oocytes retrieved and 2PN than the other group. There was no discernible difference between the two groups in terms of MII oocytes, fertilization rate, cleavage rate, or good-quality blastocyst rate. Based on the clinical outcome, there was a more significant percentage of frozen embryo transfer 75.00%) in the YA group than in the AA group (70.00%), with an OR = 0.40 (*p*-value = 0.09; 95%CI 0.14 to 1.15). In addition, there was a marginal difference in the chemical pregnancy rate between the YA and the AA group (56.67% vs. 34.48%; OR = 0.51, *p*-value = 0.162; 95%CI 0.20 to 1.30) (Table 2).

### 3.3. Factors Affect Mitochondrial Mean Fluorescence Intensity (MFI)

The factor affecting the MFI was being a woman ≥ 38 years of age (*p*-value = 0.005; 95%CI −111.4 to −19.97), total gonadotropin dosage (*p*-value = 0.006; 95%CI −0.08 to 0.01), and type of ovarian triggering drug, particularly gonadotropin-releasing hormone agonist (GnRHa) (*p*-value = 0.006; 95%CI 36.46 to 210.06). There were no significant differences in the proportions of those with BMI ≥ 25 kg/mm^3^, polycystic ovarian syndrome (PCOS), endometrioma, previous history of ovarian surgery, number of oocytes retrieved, and number of 2PN. After the multivariate regression analysis, the only persisting significant variable was women ≥ 38 or older (*p*-value = 0.014). (Table 3).

### 3.4. Factors Associated with Good Blastocyst Quality

In the multivariate analyses, three characteristics were shown to be significantly associated with high-grade blastocyst quality: progestin-primed ovarian stimulation procedure (OR = 2.14; *p*-value = 0.038, 95%CI = 1.04 to 4.42), male age (mean age ± SD = 38.26 ± 5.13; OR =0.91, *p*-value = 0.03; 95%CI 0.86 to 0.96), and aberrant semen parameter (OR = 0.56; *p*-value = 0.047, 95%CI 0.32 to 0.99). However, in the mixed multivariate analysis, only male age was significantly associated with good blastocyst quality (OR = 0.91, *p*-value = 0.008; 95 percent CI 0.85 to 0.97). Nonetheless, other factors, such as BMI, total gonadotropin dosage, trigger medication, oocyte and MII numbers, and semen analysis, had no effect on blastocyst quality. (Table 4).

## 4. Discussion

Increasing age causes ovarian aging, not only in the quantity but also in the quality of oocytes [27]. Mitochondria are crucial factors involved in ovarian aging. Previous studies have reported that in women over 38 years of age, granulosa cells have higher levels of mitochondrial DNA (mtDNA) deletion, damaged mitochondria, and abnormal gene expression [14,25]. The mitochondrial membrane potential reflects mitochondrial activity in humans [28]. Moreover, a mouse study found similar MFI, ATP, and mtDNA contents in granulosa cells [29]. Thus, this study focused on the mean MFI and was considered to represent others.

We conducted a multivariate analysis to determine the difference in the MFI between the YA and AA groups. We found that the mean MFI significantly decreased in the AA group at a mean difference of 65.71 (*p*-value = 0.013). This result is consistent with that of a retrospective study by Lu et al. Their results revealed that the MFI in CCs of advancing-age women was significantly lower than that of young women [24]. Another observational study demonstrated that mtDNA copy number and mitochondrial activity in GCs were significantly negatively correlated with advancing-age groups (β = −0.373, *p* = 0.005) [30]. Moreover, women with decreased ovarian reserves may have impaired oocyte competence due to mitochondrial biogenesis in cumulus cells [15].

Recent studies have found that some factors may interfere with mitochondrial activity and biogenesis in CCs. We also analyzed the association between the MFI and other factors. However, we found only a significant association between the MFI and women aged ≥ 38 years in univariate and multivariate analyses. In contrast to the study by Gorshinova et al., the results showed that obese women had lower mitochondrial activity than those with normal BMI [31]; our study did not show this association. In addition, a previous study revealed that the level of FSH and LH in follicular fluid from granulosa cell proliferation was linked to follicular growth and maturation [32]. Accordingly, these might be linked to the total gonadotropin dosages. Our study also found that a lower mean MFI was associated with higher total gonadotropin dosage in the univariate analysis. This result could be explained by the total gonadotropin dosage corresponding to age and ovarian reserve. However, we did not find any statistical significance after a multivariate regression analysis.

Based on the basic knowledge of folliculogenesis, the quantity of mitochondria influences oocyte development by providing them with energy substrates. It has been proposed that mitochondrial activity in CCs is a key factor in fertilization and embryogenesis [33]. These findings are consistent with current reports. The outcomes showed higher mtDNA content in CCs associated with good-quality embryos [17,18]. Unfortunately, in our study, blastocyst quality was not correlated with mitochondrial activity. This is consistent with the study by Martínez-Moro et al. These findings did not demonstrate a relationship between the mtDNA content of CCs and oocyte developmental capacity [20]. According to our findings, two recent publications [34,35] found no relationship between mtDNA in human CCs and morphological characteristics on Day 3 or subsequent implantation potential, oocyte maturity, fertilizability, embryo quality, or pregnancy rates. Although the cause of this discrepancy is unknown, it could be due to variations in mtDNA analysis or the use of disparate criteria to assess embryo developmental potential. Using morphokinetic criteria, the mtDNA content of human CCs was found to be indirectly correlated with the capability for embryonic development. From a biological perspective, this relationship is fascinating; however, it provides no more explanation for embryo selection than conventional morphological selection [36].

The authors also examined the association between these factors and embryo quality. Our findings indicated that the only male age factor linked with improved embryo quality was a lower mean age (38.26 ± 5.13 years). This is consistent with a previous study’s findings that advanced paternal age was associated with poor DNA integrity and low embryo quality [37,38,39]. It is still unclear how male factors affect early development, making the association remarkable. The influence of males on early development may be clarified by more research on APA and other factors such as medication, alcohol use, smoking, food, and other variables.

### Strengths and Limitations

Unlike most previous studies, the current study included an advancing-age group with poor and normal ovarian reserves. Furthermore, we included women who received progestin-primed ovarian stimulation (PPOS) and dual ovarian triggers, the two most recent and widely used ovarian stimulation protocols. The data showed that the PPOS protocol was likely to be associated with higher fertilization and embryo quality when compared with the antagonist protocol, but this was not statistically significant after the mixed multivariate analysis. Furthermore, we investigated the impact of age on the chemical pregnancy rate. Although the chemical pregnancy rate in the YA group was twice that in the AA group, the result was not statistically significant due to the small sample size.

This study had some limitations. First, the purpose of this observational study was to investigate mitochondrial activity in CCs only. Due to the small number of CCs, this study was unable to measure mitochondrial activity using other parameters in the same person. This, when combined with other tests, may help to produce more accurate results. Future studies using appropriate procedures are required, as this could impact ovarian maturation, fertilization, or embryo development. Due to a small sample size, a larger population should be needed to confirm the association of factors affecting the MFI and blastocyst quality.

Based on the results of this study, mitochondria in CCs showed decreased activity in the advancing-age group, and the chemical pregnancy rate also trended lower in older age. It may be advantageous to implement this process during IVF; measuring CC mitochondrial activity could be an additional method to predict the implantation rate.

## 5. Conclusions

Advancing age decreased mitochondrial activity in CCs, decreased the number of oocytes retrieved, and decreased the number of 2PN, but it did not affect blastocyst quality. Abnormal SA and PPOS protocol may be associated with blastocyst quality. However, only male age was likely to be a predictor of high-grade blastocyst quality.

## Figures and Tables

**Table 1:** Baseline characteristics.

	Young Age Group (Age < 38) (*n* = 40)	Advancing Age (Age ≥ 38) (*n* = 40)	*p*-Value
Age; years (Mean ± SD)	32.92 ± 2.54	40.92 ± 2.28	<0.001 *
BMI (kg/m^2^)	23.03 ± 3.77	23.65 ± 3.65	0.456
Infertility (%) - Primary - Secondary	26 (65.00) 14 (35.00)	25 (62.50) 15 (37.50)	0.816
Duration of infertility; years (Mean ± SD)	5.75 ± 3.40	7 ± 3.91	0.131
Male age; years (Mean ± SD)	37.35 ± 5.42	42.27 ± 5.03	<0.001 *
Cause of infertility (%) - Uterine anomalies - Tubal factors - Male factors - Multiple factors - Unknown	0 10 (25.00) 8 (20.00) 6 (15.00) 16 (40.00)	4 (10.00) 5 (12.50) 12 (30.00) 6 (15.00) 13 (32.50)	0.157
Ovarian reserve (%) - Normal - Poor	33 (82.50) 7 (17.50)	15 (37.50) 25 (62.50)	<0.001 *
AFC	9.15 ±4.35	4.55 ± 2.20	<0.001 *
Stimulation protocol (%) - Antagonist - PPOS	34 (85.00) 6 (15.00)	29 (72.50) 11 (27.50)	0.172
Total Gn dosages, IU (Mean ± SD)	2540.12± 619.59	3137 ± 736.99	<0.001 *
Total day of stimulation (Mean ± SD)	10.22 ± 0.92	10.20 ± 0.91	0.903
Type of ovarian triggering drug - Recombinant hCG (Ovidrel^®^) - Gonadotropin-releasing hormone agonist (Decapeptyl^®^) - Dual (Recombinant HCG; Ovidrel^®^ plus Gonadotropin-releasing hormone agonist; Decapeptyl^®^)	3 (7.50) 7 (17.50) 30 (75.00)	8 (20.00) 3 (7.50) 29 (72.50)	0.163

BMI: Body mass index; Gn: Gonadotropin; LOC: laparoscopic ovarian cystectomy; PPOS: Progestin- primed ovarian stimulation. * Statistically significant *p*-value 0.05.

**Table 2:** ICSI outcomes between groups.

	Young Age (Age < 38) (*n* = 40)	Advancing Age (Age ≥ 38) (*n* = 40)	Mean Difference	*p*-Value (95% CI)
MFI of CCs (Mean ± SD)	228.39 ± 121.38	162.68 ± 79.87	65.71	0.005 * (19.97, 111.45)
Number of oocytes retrieved (Mean ± SD)	16.3 ± 9.70	8 ± 5.04	8.3	<0.001 * (4.85, 11.74)
MII oocytes (%)	80.44 ± 13.45	81.83 ± 16.39	−1.38	0.679 (−8.06, 5.28)
Number of 2PN (Mean ± SD)	9.6 ± 5.93	4.77 ± 3.07	4.82	<0.001 * (2.72, 6.929)
Fertilization rate (%)	75.37 ± 15.19	76.59 ± 22.85	−1.21	0.780 (−9.85, 7.42)
Cleavage rate (%)	98.27 ± 6.90	96.40 ± 13.55	1.87	0.438 (−2.91, 6.66)
Blastocyst rate (%)	56.99 ± 23.26	62.55 ± 29.93	−5.56	0.356 (−17.49, 6.37)
Good-quality blastocyst rate (%)	23.56 ± 23.71	19.22 ± 28.65	4.33	0.463 (−7.37, 16.04)
Frozen embryo transfer (%)	30 (75.00)	28 (70.00)	0.40 ^a^	0.090 (0.14, 1.15)
Chemical pregnancy (%)	17 (56.67)	10 (34.48)	0.51 ^a^	0.162 (0.20, 1.30)

CCs: Cumulus cells; ICSI: Intracytoplasmic sperm injection; IVF: In vitro fertilization; MFI: mean fluorescence intensity; PN: pronuclei. ^a^ Odd ratio (OR). * Statistically significant *p*-value 0.05.

**Table 3:** Factors affect mean fluorescence intensity (MFI).

Factors	MFI	
	Univariate Analysis *p*-Value (95%CI)	Multivariate Analysis *p*-Value (95%CI)
Age ≥ 38 years	0.005 * (−111.45, −19.97)	0.014 (−121.00, −14.30) *
Obesity (BMI ≥ 25 kg/m^2^)	0.850 (−49.40, 59.84)	0.712 (−42.64, 62.01)
Polycystic ovarian syndrome (PCOS)	0.591 (−160.56, 92.04)	0.198 (−219.82, 46.34)
Endometrioma	0.467 (−115.89, 53.68)	0.524 (−114.87, 59.01)
Previous ovarian surgery (unilateral LOC)	0.113 (−384.47, 41.30)	0.055 (−408.69, 4.19)
Total Gn dosages	0.006 * (−0.08, −0.01)	0.198 (−0.01, 0.01)
Type of ovarian triggering drug - Recombinant hCG (Ovidrel^®^) - Gonadotropin-releasing hormone agonist (Decapeptyl^®^) - Dual (Recombinant HCG; Ovidrel^®^ plus Gonadotropin-releasing hormone agonist; Decapeptyl^®^)	Ref 0.006 * (36.46, 210.06) 0.867 (−70.74, 59.74)	Ref 0.133 (−21.44, 158.22) 0.171 (−120.50, 21.77)
Number of oocytes retrieved	0.897 (−2.58, 2.95)	0.557 (−4.19, 7.71)
Number of 2PN	0.991 (−4.60, 4.55)	0.353 (−14.41, 5.22)

BMI: Body mass index; Gn: Gonadotropin; LOC: laparoscopic ovarian cystectomy; MFI: mean fluorescence intensity; PN: pronuclei. * Statistically significant *p*-value 0.05.

**Table 4:** Characteristics and blastocyst quality outcome.

	Good Quality (*n* = 83)	Fair or Poor Quality (*n* = 246)	Univariate Analysis OR (*p*-Value; 95%CI)	Multivariate Analysis OR (*p*-Value; 95%CI)	Mixed Multivariate Analysis OR (*p*-Value; 95%CI)
Age ≥ 38	31 (37.35)	87 (35.37)	1.08 (0.745; 0.65–1.82)	1.30 (0.487; 0.61, 2.75)	1.30 (0.535; 0.56, 3.04)
BMI	23.99 ± 3.91	23.83 ± 3.74	1.01 (0735; 0.94–1.07)	1.01 (0.671; 0.94, 1.09)	1.01’ (0.835, 1.10)
Total Gn dosage	2735.24 ± 704.58	2708.17 ± 684.86	1.00 (0.757; 0.99–1.00)	1.00 (0.832; 0.99, 1.00)	1.00 (0.740; 0.99, 1.00)
Protocol					
- Antagonist	64 (77.11)	210 (85.37)	Ref	Ref	Ref
- PPOS	19 (22.89)	36 (14.63)	1.73 (0.084; 0.92–3.22)	2.14 (0.038; 1.04, 4.42) *	2.20 (0.060; 0.96, 5.02)
Trigger drugs - Recombinant hCG (Ovidrel^®^) - Gonadotropin-releasing hormone agonist (Decapeptyl^®^) - Dual (Recombinant HCG; Ovidrel^®^ plus Gonadotropin-releasing hormone agonist; Decapeptyl^®^)	5 (6.02) 13 (15.66) 65 (78.31)	17 (6.91) 27 (10.98) 202 (82.11)	Ref 1.63 (0.420; 0.49–5.41) 1.09 (0.865; 0.38–3.08)	Ref 2.84 (0.155; 0.67, 12.03) 1.08 (0.887; 034–3.38)	Ref 3.46 (0.137; 0.67, 17.76) 1.10 (0.881; 0.31, 3.84)
Male age	38.26 ± 5.13	39.95 ± 5.96	0.94 (0.023; 0.90, 0.99) *	0.91 (0.003; 0.86, 0.96) *	0.91 (0.008; 0.85, 0.97) *
Abnormal SA	25 (30.12)	102 (41.46)	0.60 (0.068; 0.35–1.03)	0.56 (0.047; 0.32, 0.99) *	0.56 (0.087; 0.29, 1.08)
Number of oocytes retrieved	16.19± 9.46	17.64 ± 11.82	0.98 (0.311; 0.96,−1.01)	0.99 (0.821; 0.96, 1.02)	0.99 (0.956; 0.95, 1.04)
Percent MII oocytes	80.91 ± 12.30	80.39 ± 13.84	1.00 (0.763; 0.98, 1.02)	1.00 (0.760; 0.99, 1.00)	0.99 (0.793; 0.97, 1.02)
MFI	195.47 ± 115.03	192.19 ± 97.88	1.00 (0.800; 0.99, 1.00)	1.00 (0.189; 0.9, 1.001)	0.99 (0.222; 0.99, 1.00)

BMI: Body mass index; Gn: Gonadotropin; MFI: mean fluorescence intensity; PPOS: Progestin- primed ovarian stimulation; SA: semen analysis * Statistically significant *p*-value 0.05.

## Data Availability

The data presented in this study are available on request from the corresponding author due to ethical reason.
